# Early career researcher responses to 2025 United States federal science funding disruptions: a rapid-response pilot survey

**DOI:** 10.3389/fsoc.2026.1790840

**Published:** 2026-07-20

**Authors:** Crystal Hammond, John Patrick Flores, Siara Rouzer, Kassandra Fernandez, Amy Ralston, Adriana Bankston

**Affiliations:** 1 School of Public Policy, University of California, Riverside, Riverside, CA, United States; 2 The STEM Advocacy Institute, Boston, MA, United States; 3 Science for Good, Washington, DC, United States; 4 Curriculum of Bioinformatics and Computational Biology, Department of Genetics, University of North Carolina (UNC) Chapel Hill School of Medicine, Chapel Hill, NC, United States; 5 Department of Neuroscience and Experimental Therapeutics, Texas A&M University College of Medicine, Bryan, TX, United States; 6 Department of Engineering Education, University of Florida, Gainesville, FL, United States; 7 Department of Physics and Astronomy, University of California, Irvine, Irvine, CA, United States; 8 US House of Representatives, Washington, DC, United States

**Keywords:** academic research, early career researcher (ECR), federal policy making, science funding cuts, STEM workforce development, Trump administration

## Abstract

**Introduction:**

Scientific research drives national progress and innovation, but abrupt federal funding disruptions can destabilize scientific training at critical career stages and push talented researchers out of the workforce. Actions by the Trump administration in early 2025 sent shockwaves across academic research in the United States (U.S.). Federal science budgets were significantly reduced, hundreds of research grants were abruptly frozen or terminated, and politically appointed agency leaders overrode peer-reviewed funding decisions.

**Methods:**

We conducted pilot, rapid-response surveys to capture quotes, stories, and quantitative data in order to understand how these seismic policy shifts impacted early career researchers at American universities.

**Results:**

Our results showed that the majority of respondents were PhD students and early career researchers at varying stages of training, across a range of institutions and disciplines, including different types of sciences, with the largest percentage of respondents from the life sciences. Respondents described threats to the continuity of their research and careers due to funding cuts at major federal science agencies including the National Institutes of Health (NIH) and the National Science Foundation (NSF), and reported concerns about long-term job prospects and broader systemic issues in academia. Many respondents also indicated that these disruptions led them to consider leaving research altogether or pursuing scientific careers outside the U.S. Reported concerns spanned the undergraduate, graduate, and postdoctoral researcher levels. While universities provided some resources of support, respondents emphasized the need for Congress to protect, stabilize, and restore science funding.

**Discussion:**

Robust and sustained funding support for early career researchers is imperative for developing a strong science, technology, engineering, and math (STEM) workforce and maintaining American leadership in research and innovation. Overall, this pilot study provides early evidence that reductions in federal science funding may disrupt training pathways, erode confidence in research careers, and contribute to lasting losses from the future research workforce in the United States.

## Current landscape

Sustained federal research funding supports the development of a strong pipeline, which drives forward scientific innovations. In early 2025, a surge of executive orders and the Trump administration's FY 2026 budget proposal sent shockwaves across the academic research landscape, targeting areas ranging from National Institutes of Health (NIH) extramural funding to climate science and diversity, equity, and inclusion (DEI)-related programs (Glenza, 2025; Waldman, 2025). Federal science budgets were reduced by double-digit percentages, hundreds of research grants were abruptly frozen or terminated, and politically-appointed agency leaders overrode peer-reviewed funding decisions (Palmer, 2025a; Bhatia et al., 2025). Together, these actions led to substantial funding uncertainty for research institutions, laboratories, and scientists across the United States (U.S.).

These disruptions quickly affected university research activity, hiring, and staffing. Early March 2025 marked a turning point for researchers nationwide as federal proposals began reducing science funding across agencies (Bankston, 2025). On March 10, the University of Pennsylvania announced a campus-wide hiring freeze and a 5% cut in non-compensation expenses (Graham, 2025). By March 19, University of California (UC) System then-President Michael Drake had ordered a system-wide freeze on new faculty and staff searches alongside other cost-saving measures, while also signaling a more cautious approach to spending in response to federal funding cuts (Ward, 2025). On April 8, Northwestern University paused hiring and reduced discretionary spending by 10 percent (Schill et al., 2025). Although these examples represent only a subset of institutional responses, they illustrate the Trump administration's negative impact on recruiting and retaining STEM talent in U.S. laboratories.

The effects of these policy changes were also evident in programs that support the next generation of STEM researchers. In April 2025, funding for the National Science Foundation (NSF) Graduate Research Fellowship Program (GRFP) was cut roughly in half, from about 2,000 awards to 1,000 (Mervis, 2025). NSF also removed eligibility for second-year PhD students to apply for the GRFP, a program critical to developing U.S. STEM talent (Langin, 2025) and this could impair the ability of students to join labs after their rotations. Funding to several NIH supported programs, including the Institutional Research and Academic Career Development Award (IRACDA) program, was also cut by the Trump administration (SCIMaP, 2025) and later restored by federal courts (Rahal, 2025). Within the UC System, president at the time of publication president James Milliken later reversed an earlier decision to end financial support for hiring postdoctoral fellows in the UC President's Postdoctoral Fellowship Program (Whitford, 2025). Although the NSF GRFP award number was eventually partially restored by funding an additional 500 NSF GRFP grants (National Science Foundation, 2025), and the NSF funded 2,500 Graduate Research Fellowships for the 2026–2027 academic year (National Science Foundation, 2026), this disruption in funding at major federal science agencies including NSF has undermined key training and career-development pathways.

In May 2025, the Trump administration released its FY 2026 budget proposal calling for a 40 percent cut to the NIH and a 55 percent cut to the NSF (The White House, 2026; Agarwal, 2025), freezing new grants and bringing many research projects to a sudden halt (The White House, 2026). These impacts were documented by several organizations, including the Association of American Universities (AAU), highlighting the breadth of the proposed funding cuts across federal science agencies (Agarwal, 2025). While these are only a few examples, they demonstrate how quickly federal actions can halt scientific progress across federal agencies and research institutions nationwide, with negative downstream consequences for training and STEM workforce development (Ragland and Bedekovics, 2025).

Despite widespread reporting on grant terminations, hiring freezes, and agency-level policy changes, there remains limited empirical evidence on how early career researchers themselves are experiencing these disruptions in real time. This gap is important because early career researchers occupy especially vulnerable positions within the academic labor structure: they often depend on grant-funded salaries (U.S. Government Accountability Office, 2026), fellowships, training programs, and institutional hiring pipelines, yet have relatively limited power to absorb sudden disruptions. As universities have begun to cut PhD admissions (Mao and Paulus, 2025; Witze, 2025), early career researchers are increasingly seeking opportunities elsewhere due to rising job losses and federal grant terminations at U.S. institutions. Simultaneously, other nations are heavily recruiting STEM talent away from the U.S. (Larson et al., 2025; González, 2025; Jack, 2025), drawing highly-skilled scientists abroad through substantial investments in innovation and research infrastructure (Mason, 2025). Furthermore, staff reductions and job losses among scientific experts at U.S. federal agencies, including the NSF, will significantly undermine national leadership in science and technology by weakening support for federally-funded research, education, and diversity initiatives (Lambert, 2025). Additionally, cuts to indirect costs at the NIH carry severe consequences for American science and innovation (Sinclair et al., 2025). Without sustained federal investment and the expert workforce needed to administer these programs, early career researchers may face growing barriers to initiating, maintaining, and advancing independent research careers in U.S. laboratories.

## Context and methods

This work was performed as part of The Stem Advocacy Institute (SAi), a Massachusetts-based non-profit organization specifically operating as the Sai Resident Collective (SRC), an experimental incubator and community of residents working to strengthen the connections between science and society. Developed by the Civic Science Media Lab, this network is built on a residency concept aiming to strengthen connections and encourage individuals who show exceptional promise and creativity to advance entrepreneurial and scholarly projects. As an SRC resident, Adriana Bankston leads the Biomedical Workforce & Policy Research Group (Bankston Group) whose goal is to cultivate a robust STEM workforce, and which includes as members early career scientists and STEM graduates who are driving research forward and represent the future of the field (Bankston Group, 2022). In order to understand how seismic policy shifts resulting from the Trump administration's actions have been experienced on the ground, especially by early career researchers at a critical juncture in their scientific paths, the Bankston Group conducted an initial rapid-response survey to collect qualitative accounts and to assess whether respondents had taken any steps toward advocacy for research (Survey 1, Supplementary Appendix). We then conducted a second, more comprehensive, data-driven survey to examine the impacts of federal funding cuts on early career researchers and on the broader research workforce in U.S. universities (Survey 2, Supplementary Appendix). Figures were originally published in a preprint, which additionally contains more information about how early career researchers organized nationwide to fight back against the Trump administration (Hammond et al., 2025). In the context of broader impacts of this work, both surveys were conducted in collaboration with the non-profit Science for Good (Science for Good, 2025). The Goucher College Institutional Review Board (IRB) determined this research project to be exempt from IRB review (EXEMPT #20141751).

### Data collection

Both surveys were administered online using Google Forms between May 23 and June 6, 2025. This limited time frame was chosen to capture time-sensitive responses from early career researchers while the effects of the Trump administration's actions on federal research funding were still unfolding. Survey 1 was brief and qualitative. It collected respondents' institutional affiliations and provided an opportunity to describe how they had been affected, as well as any advocacy actions they had taken. The brevity in timing of this first survey was intended to facilitate rapid-response data collection during a period of acute disruption. Survey 2 was longer and included both quantitative and qualitative items. It collected additional demographic information and more detailed data on the impacts of and concerns related to federal research funding cuts among early career researchers following the Trump administration's policy actions on science and education. This approach was intended to provide a clearer picture of how early career researchers across disciplines and institutions nationwide were experiencing these changes (Survey 2). Survey participants were recruited through academic listservs, social media, and direct outreach to contacts in universities and non-profits, including those working in career development, research, policy, and STEM pipeline diversification (Supplementary Table 1). Survey participation was voluntary, uncompensated, and opt-in anonymous with respect to personally identifiable information. Both surveys used convenience sampling, and therefore no effort was made to control for demographic representation in the dataset, and this collection method did not allow for calculating response rates.

**Table 1:** Academic institutions represented by survey respondents (Q3).

University represented by respondents	Public or private university	Carnegie classification
Arizona State University^**^	Public	R1
Baylor College of Medicine	Private	R1
Boston University	Private	R1
Emory University^**^	Private	R1
Georgetown University^**^	Private	R1
Johns Hopkins University	Private	R1
Massachusetts Institute of Technology	Private	R1
Medical College of Wisconsin	Private	R2
Medical University of South Carolina	Public	R1
Morehouse School of Medicine (graduate)^**^	Private	RCU
Oregon Health & Science University	Public	R2
University of Alaska Fairbanks^**^	Public	R2
University of California Irvine	Public	R1
University of California Los Angeles	Public	R1
University of California San Diego	Public	R1
University of Colorado Boulder	Public	R1
University of Kentucky	Public	R1
University of Minnesota Twin Cities	Public	R1
University of Pennsylvania	Private	R1
University of South Dakota	Public	R2
University of Utah	Public	R1
University of Virginia	Public	R1
Virginia Tech	Public	R1
Washington University in St. Louis	Private	R1

Respondents reported the academic institutions at which they were studying or working at the time of the survey. These institutions were then classified as public or private and assigned Carnegie research designations of R1 (Very High Research Spending and Doctorate Production), R2 (High Research Spending and Doctorate Production), or RCU (Research Colleges and Universities). This table includes self-reported institutional affiliations from both surveys, including the full list from Survey 2 and the additional institutions represented in Survey 1, indicated by (^**^).

### Data analysis

Preliminary results from Survey 1 were previously discussed in a blog post (Bankston, 2025), in which participant information was shared only with explicit consent. Additionally, due to the highly personal nature of some participant responses, full transcripts are not included in this publication in order to protect confidentiality. Quantitative data from Survey 2 were tabulated and analyzed using descriptive statistics, organized into data tables, and visualized graphically. For survey questions allowing multiple selections, response options were non-mutually exclusive, and percentages reflect the proportion of respondents selecting each option. Qualitative data from both surveys were summarized and are presented in narrative form.

## Results

In this publication, we present results from the quantitative survey (Survey 2; 30 respondents), supplemented by selected perspectives from the stories survey, including any advocacy actions taken by respondents (Survey 1; six respondents) in order to contextualize our findings. Survey 2 yielded responses on career stage, time in current position, institution, and field of study, as well as optional demographic information. It also assessed sources of federal research funding, the impacts of funding cuts on the careers and trajectories of early career researchers, concerns about future research prospects, intentions to leave academic research, institutional resources provided, and perspectives on how Congress could offer support.

### Respondent characteristics

**Figure 1:**
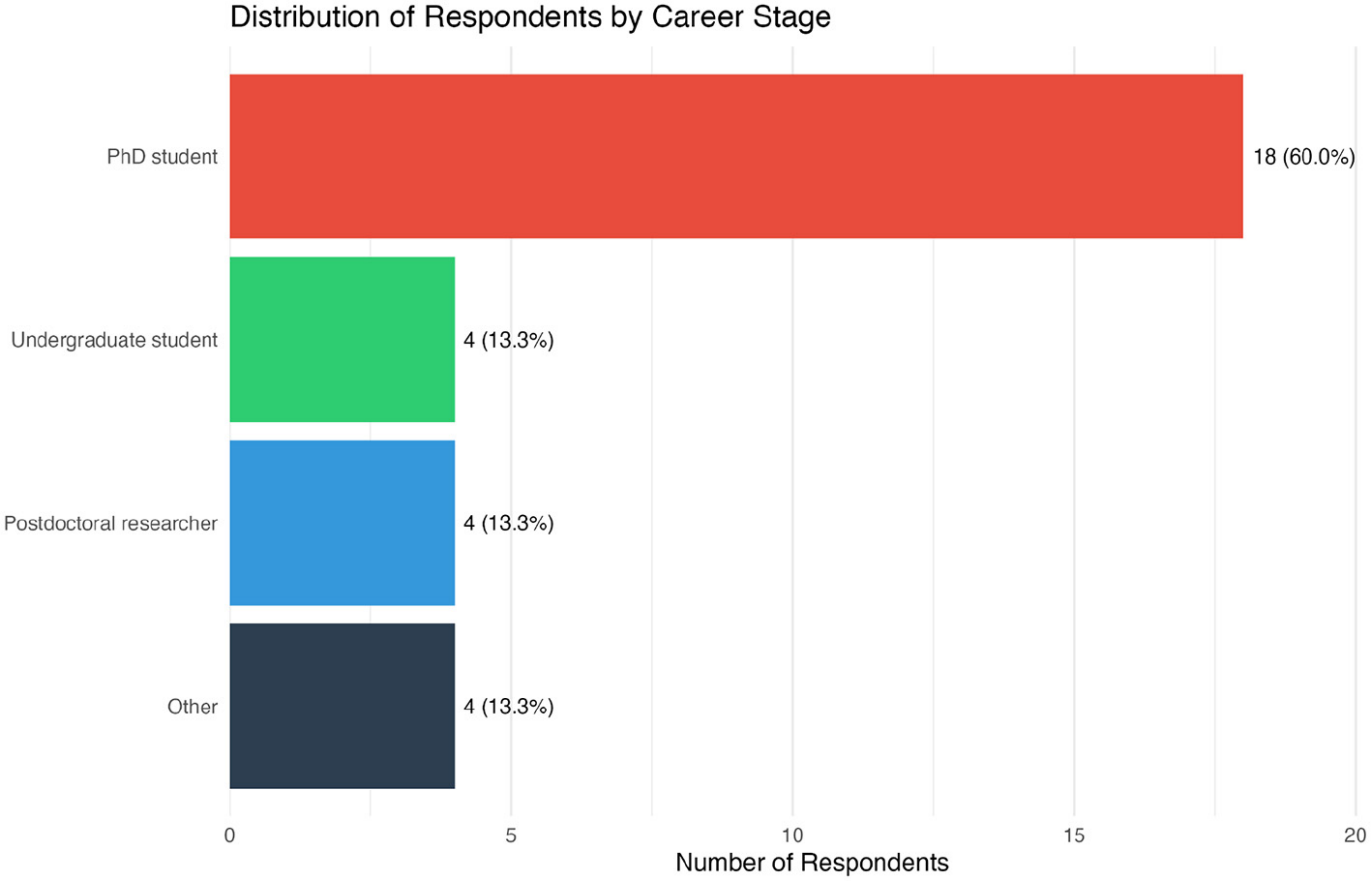
Career stage of respondents (Q1). In Survey 2, the majority of respondents were PhD students (60%), followed by undergraduate students (13.3%), postdoctoral researchers (13.3%) and respondents in the “other” category (13.3%); no respondents identified as Master's students.

**Figure 2:**
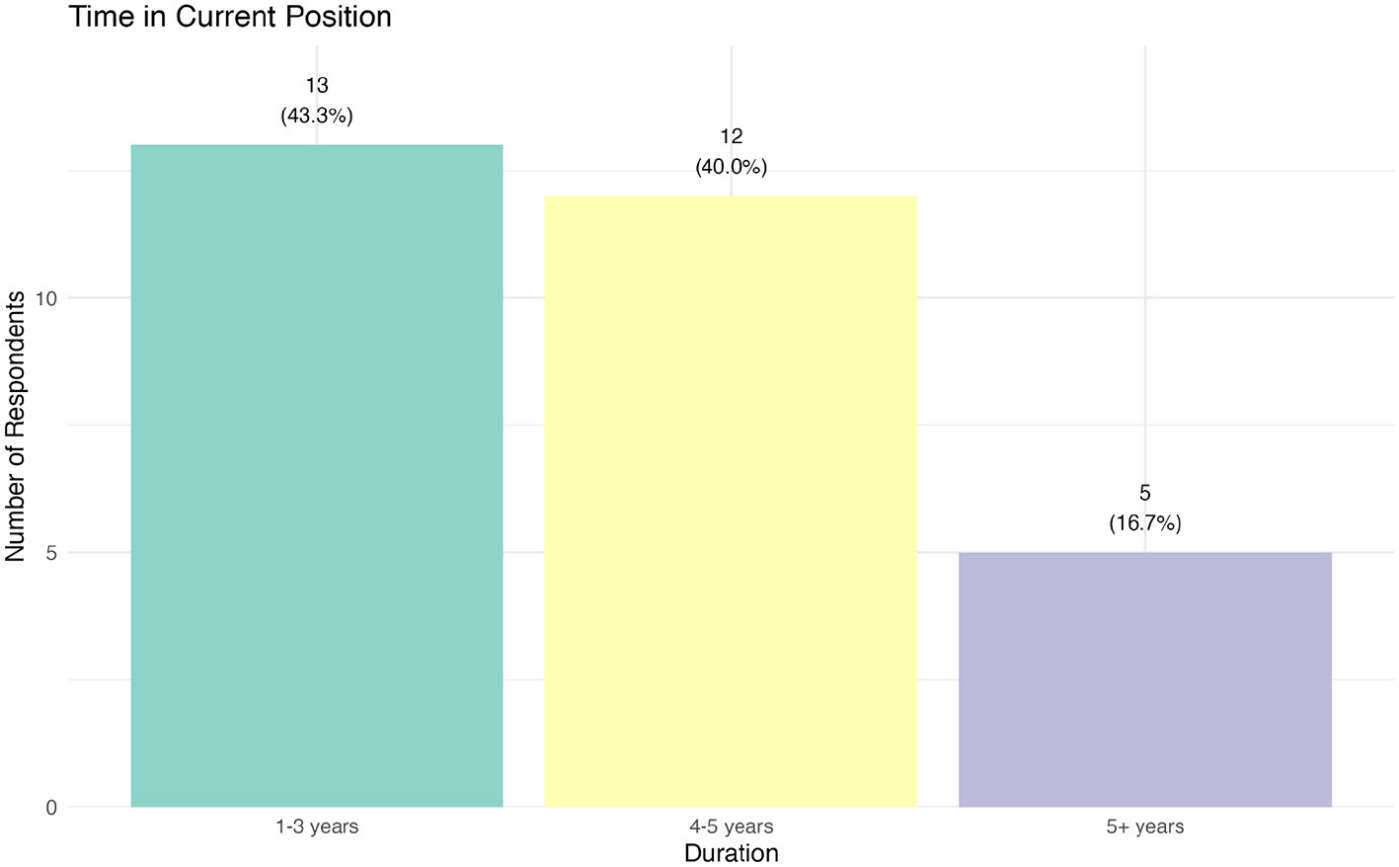
Respondents' time in current position (Q2). In Survey 2 respondents had been in their current positions for 1–3 years (43.3%), followed by 4–5 years (40%), and more than 5 years (16.7%).

For career stage (Q1), the majority of respondents were PhD students (60%), followed by undergraduate students (13.3%), postdoctoral researchers (13.3%), and respondents in the “other” category (13.3%); no respondents identified as Master's students (Figure 1). At the time of the survey, respondents had been in their current positions (Q2) for 1–3 years (43.3%), followed by 4–5 years (40%), and more than 5 years (16.7%; Figure 2). The predominance of PhD students and respondents who had been in their positions for 1–5 years is notable, given the important role of this population in the STEM workforce, and the uncertainty facing early career researchers in the current funding environment.

**Figure 3:**
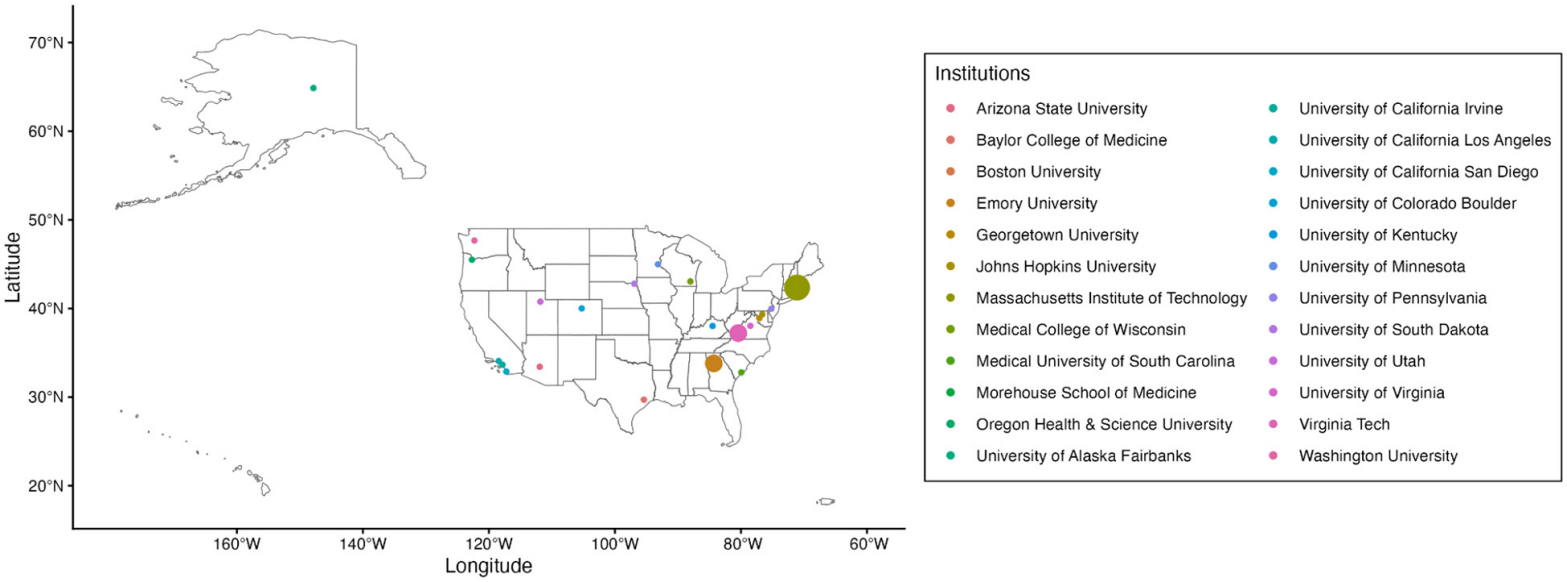
Map of academic institutions represented by survey respondents (Q3). This map displays the self-reported academic institutions listed in Table 1, based on responses from both surveys. Larger circles indicate a greater number of survey responses from a given institution.

Respondents reported the academic institution at which they were studying or working at the time of the survey (Q3), and these results are summarized in Table 1. We then classified institutions as public or private, and assigned Carnegie research designations using the Carnegie Classification of Institutions of Higher Education (American Council on Education, 2022). These classifications categorize U.S. colleges and universities based on characteristics such as degree types awarded, doctoral degree production, and research expenditures. Institutions represented in our sample fell into three categories: R1: Very High Research Spending and Doctorate Production, defined as institutions that spend at least $50 million on research and development and award at least 70 research doctorates annually R2: High Research Spending and Doctorate Production, defined as institutions that spend at least $5 million on research and development and award at least 20 research doctorates annually; and Research Colleges and Universities (RCU), defined as institutions that spend at least $2.5 million on research and development, but do not meet the criteria for R1 or R2 (American Council on Education, 2022). Based on these classifications, the majority of respondents were affiliated with R1 institutions (~67%) and public institutions (~67%; Table 1), and the largest share of institutions represented were located in the Northeastern U.S. (~43%; manually determined and calculated based on survey data; Figure 3). The predominance of respondents from R1 and public institutions may reflect patterns in survey outreach and/or a greater likelihood of participation among individuals motivated to report the impacts of federal funding changes on their research.

Using these data, we generated a map in R by geocoding the institutions reported by survey respondents (Table 1) and overlaying them as colored points on a U.S. map, with larger circles indicating a greater number of survey responses (Figure 3). Although the represented institutions were concentrated in the Northeast and Mid-Atlantic, respondents' institutions were distributed across the United States, including the South, Midwest, West, and Alaska. These responses suggest that the effects reported by early career researchers were not confined to a single region and reflect a broader national pattern.

**Figure 4:**
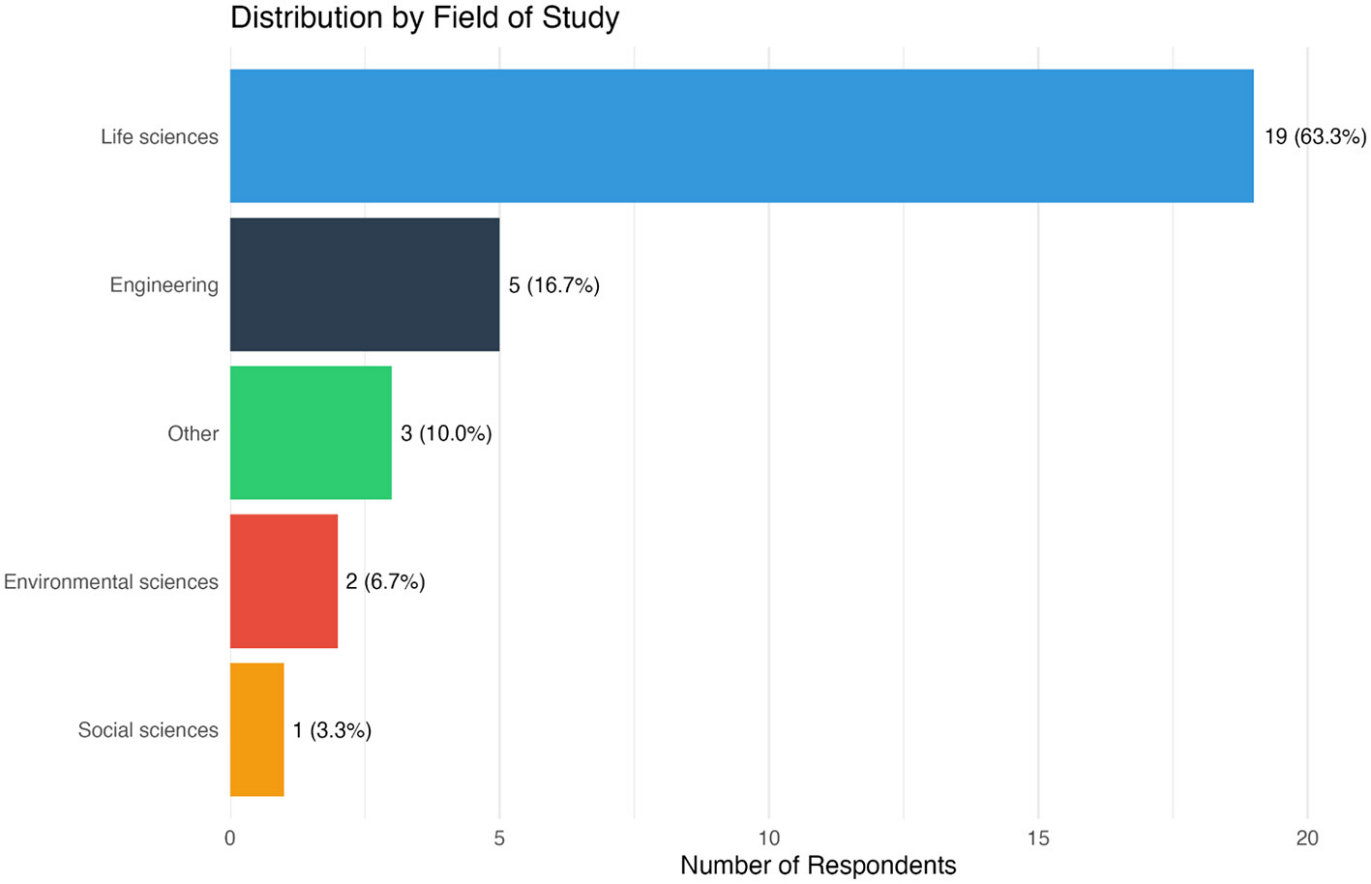
Field of study of respondents (Q4). In Survey 2, the majority of respondents were in the life sciences (63.3%), followed by engineering (16.7%), other disciplines (10.0%), environmental sciences (6.7%), and social sciences (3.3%).

For research disciplines (Q4), we used broad outreach across organizations to capture responses from early career researchers across the U.S. and across major research fields. The majority of respondents were in the life sciences (63.3%), followed by engineering (16.7%), other disciplines (10.0%), environmental sciences (6.7%), and social sciences (3.3%; Figure 4). Although this distribution was not intentionally targeted, it is consistent with the prominence of life sciences research within U.S. universities (CBRE, 2023).

**Figure 5:**
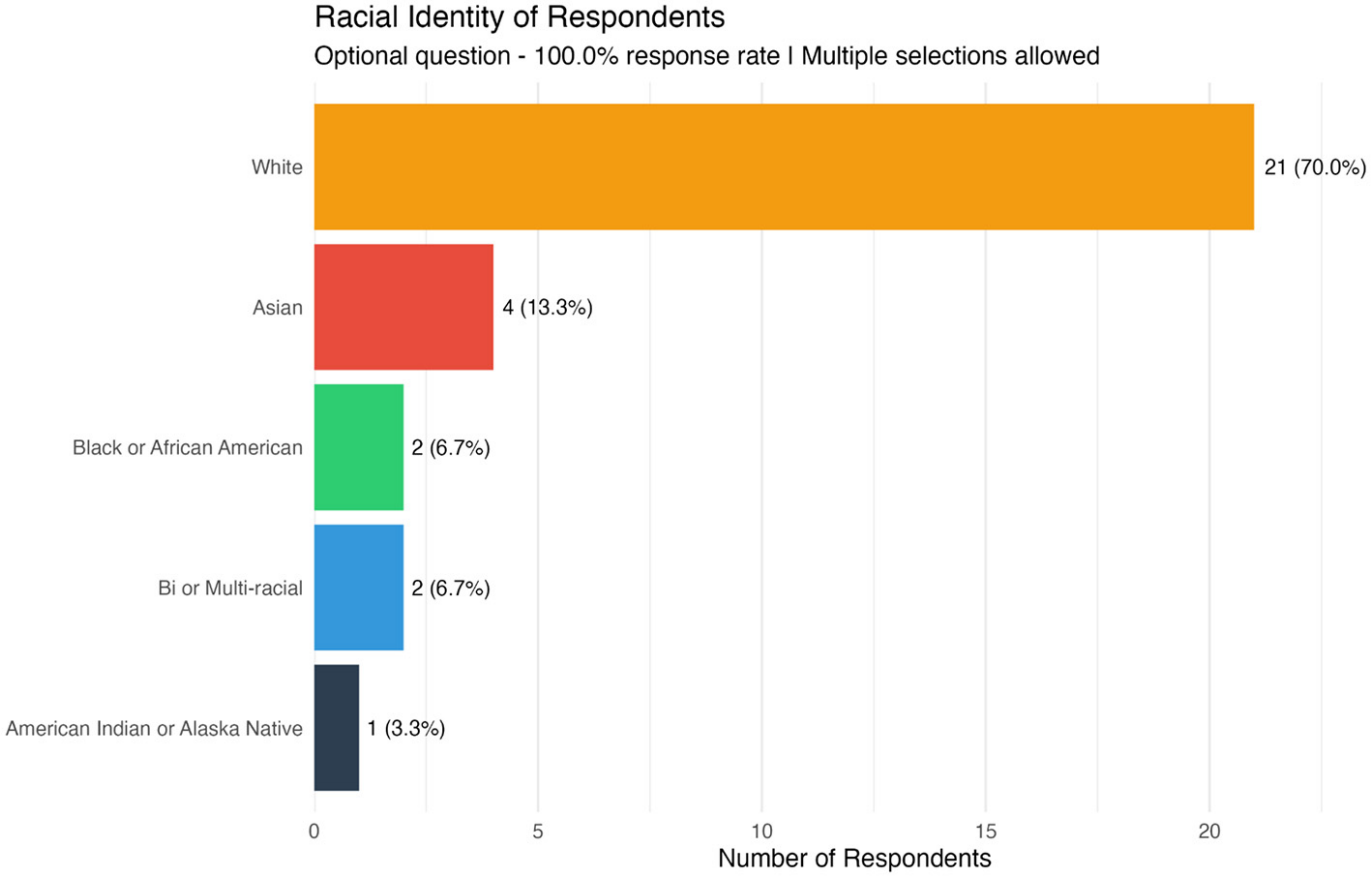
Racial identity of respondents (Q5). In Survey 2 the majority of respondents identified as White (70.0%), followed by Asian (13.3%), Black or African American (6.7%), bi- or multiracial (6.7%), and American Indian or Alaska Native (3.3%); no respondents selected Native Hawaiian or Other Pacific Islander or the “Other” category.

Although the demographics survey question was optional (Q5), all respondents provided an answer. The majority of respondents identified as White (70%), followed by Asian (13.3%), Black or African American (6.7%), bi- or multiracial (6.7%), and American Indian or Alaska Native (3.3%); no respondents selected Native Hawaiian or Other Pacific Islander or the “Other” category (Figure 5). Given the small sample size and convenience sampling design, these distributions should be interpreted with caution. The limited representation of some minoritized groups may reflect multiple factors, including differential patterns of survey reach, participation, and willingness to respond. However, the limited representation of some minoritized groups may reflect broader concerns about vulnerability, visibility, or potential retaliation in the context of federal policy changes.

**Figure 6:**
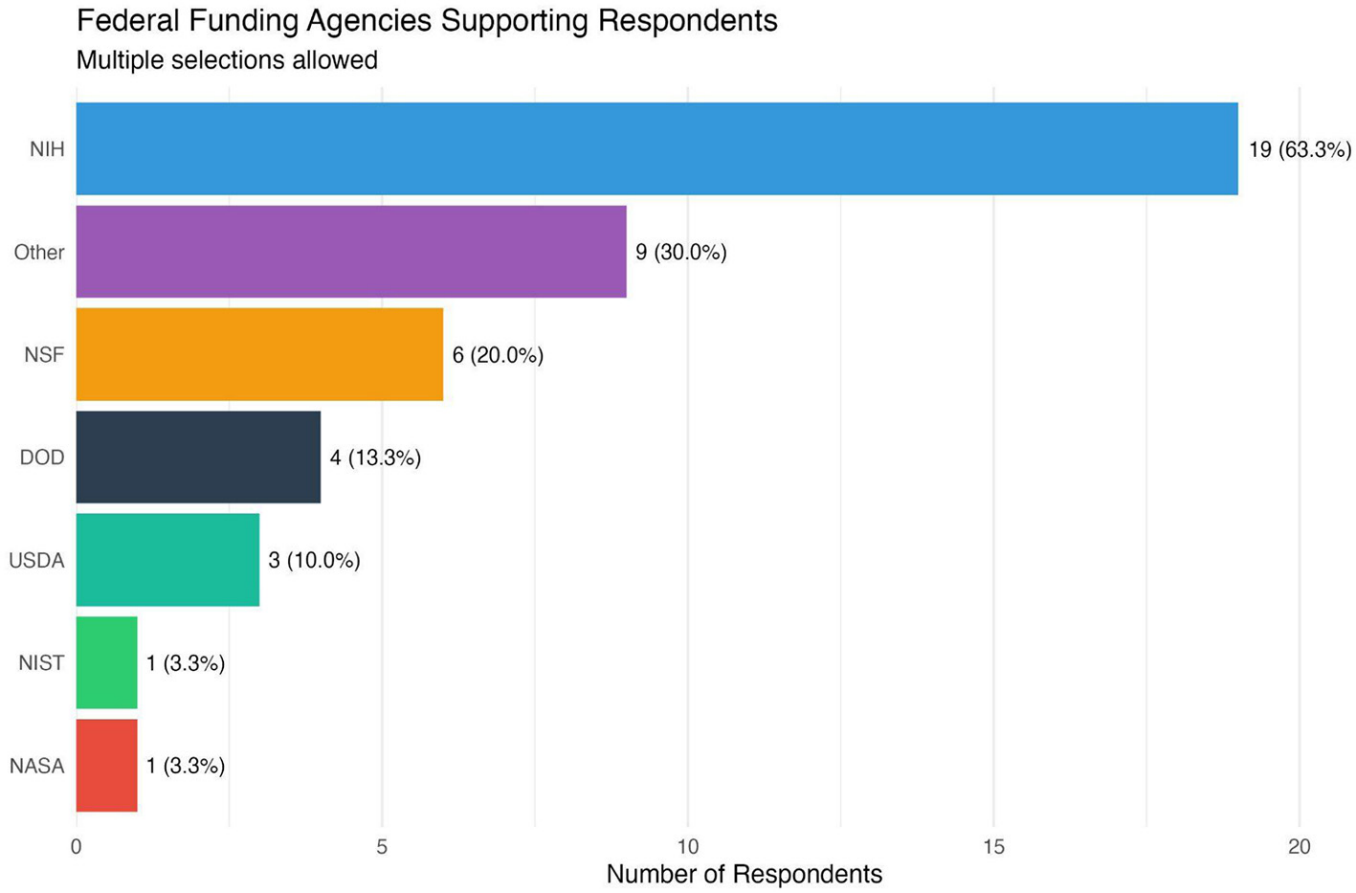
Federal funding agencies supporting respondents' research (Q6; multiple responses permitted). In Survey 2, respondents most frequently identified NIH as a source of research funding (63.3%), followed by other sources (30.0%), NSF (20.0%), the DOD (13.3%), USDA (10%), NIST (3.3%), and NASA (3.3%); no respondents selected DOE. Acronyms: NIH, National Institutes of Health; NSF, National Science Foundation; DOD, Department of Defense; USDA, US Department of Agriculture; NIST, National Institute of Standards and Technology; NASA, National Aeronautics and Space Administration; DOE, Department of Energy.

Respondents were asked which federal agencies provided support for the research they performed (Q6, multiple responses permitted). The most frequently reported funding source was the NIH (63.3%), followed by other sources (30.0%), the NSF (20.0%), the Department of Defense (DOD; 13.3%), the United States Department of Agriculture (USDA; 10.0%), the National Institute of Standards and Technology (NIST; 3.3%), and the National Aeronautics and Space Administration (NASA; 3.3%); no respondents elected the Department of Energy (DOE; Figure 6). These results highlight the central role of federal science agencies in supporting the research conducted by early career scientists in our sample.

### Funding exposure and immediate professional impacts

**Figure 7:**
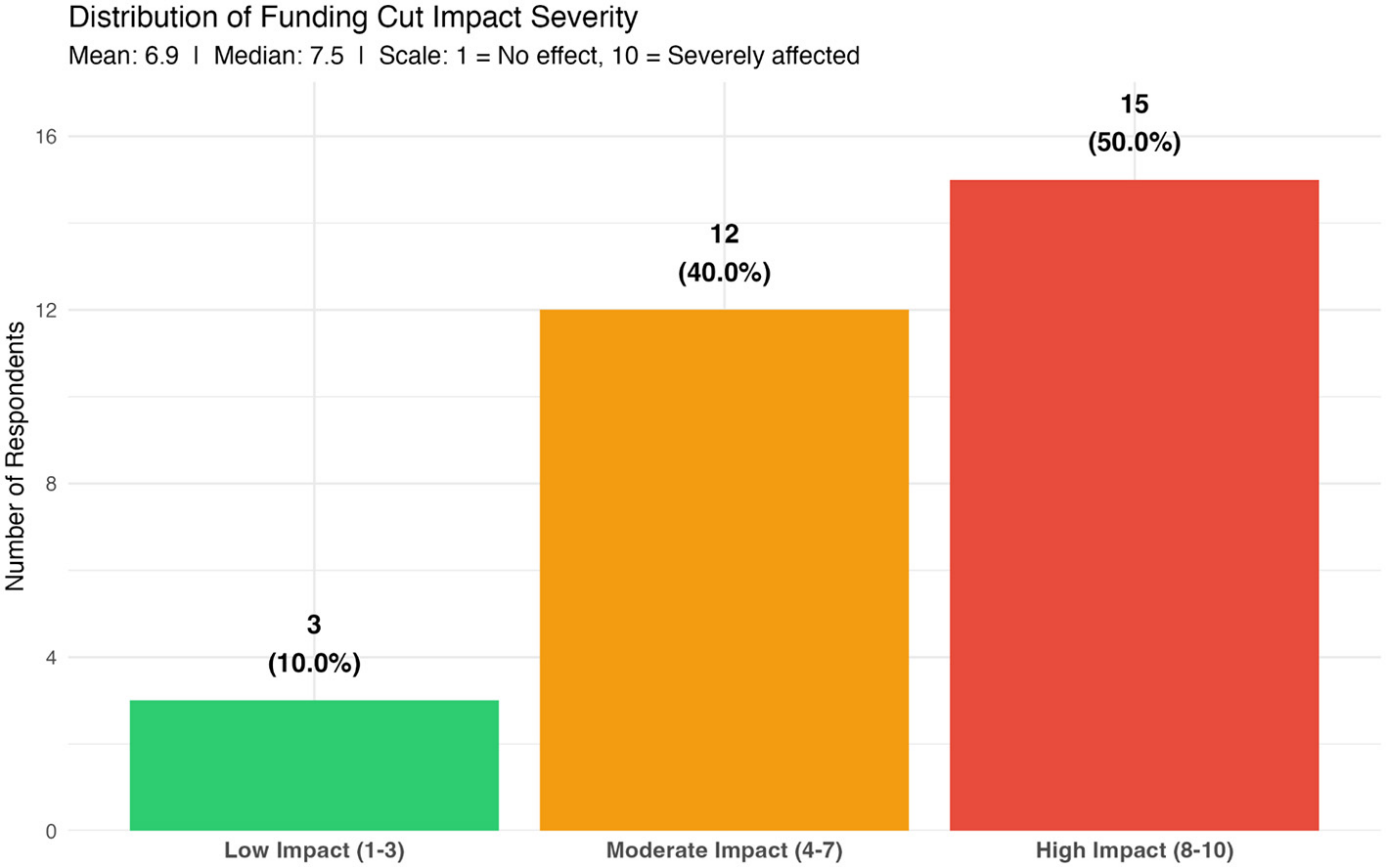
Impact severity of federal research funding cuts on respondents (Q7). In Survey 2, respondents rated the professional impact of federal research funding cuts on a 1–10 scale, where 1 indicated no effect and 10 indicated being severely affected. For visualization, responses were grouped into low impact (scores 1–3), moderate impact (scores 4–7), and high impact (scores 8–10). Respondents reported low (10.0%), moderate (40.0%), and high (50.0%) levels of impact severity.

Respondents were also asked to rate the professional effects of science funding cuts on a scale from 1 to 10, where 1 indicated no effect, 5 indicated a moderate effect, and 10 indicated a severe effect (Q7). For visualization, responses were grouped into low impact (scores 1–3), moderate impact (scores 4–7), and high impact (scores 8–10). Respondents reported low (10.0%), moderate (40.0%), and high (50.0%) levels of severity in professional impact associated with federal funding cuts (Figure 7). Although these findings should be interpreted with caution given the small sample size and convenience sampling approach, the predominance of moderate and high impact ratings suggests that funding cuts were widely perceived by respondents as professionally disruptive.

**Figure 8:**
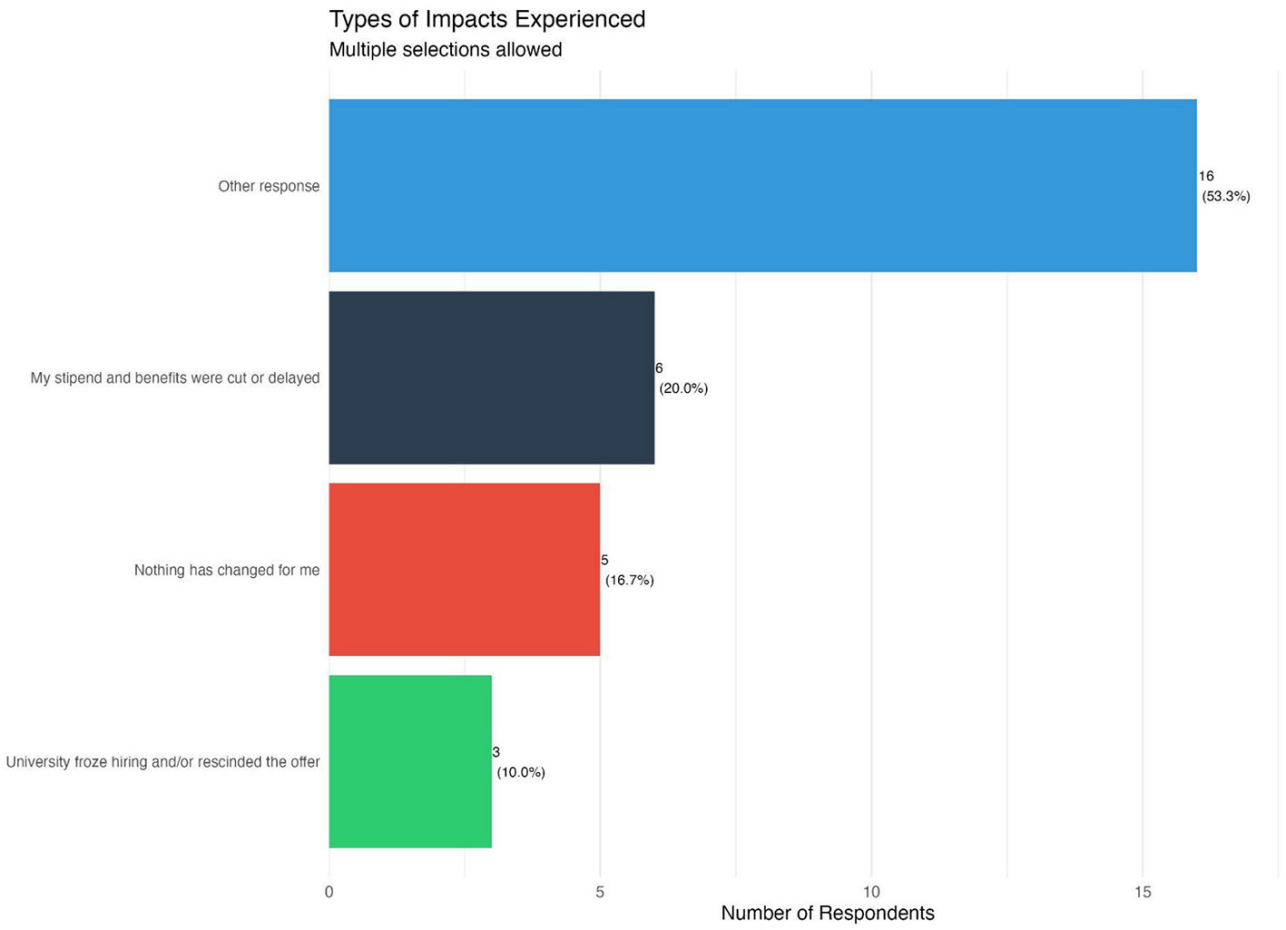
Professional impacts resulting from science funding cuts (Q8). In Survey 2, respondents most frequently reported “other” impacts on their careers, (53.3%), followed by stipend and benefit cuts or delays (20.0%), no change (16.7%), and hiring freezes and/or rescinded offers (10.0%).

When asked about professionally negative impacts experienced by early career researchers as a result of university actions (Q8), respondents most frequently selected “other” (53.3%), followed by stipend and benefit cuts or delays (20.0%), no change (16.7%), and hiring freezes and/or rescinded offers (10.0%; Figure 8).

### Anticipated consequences for training and career trajectories

**Figure 9:**
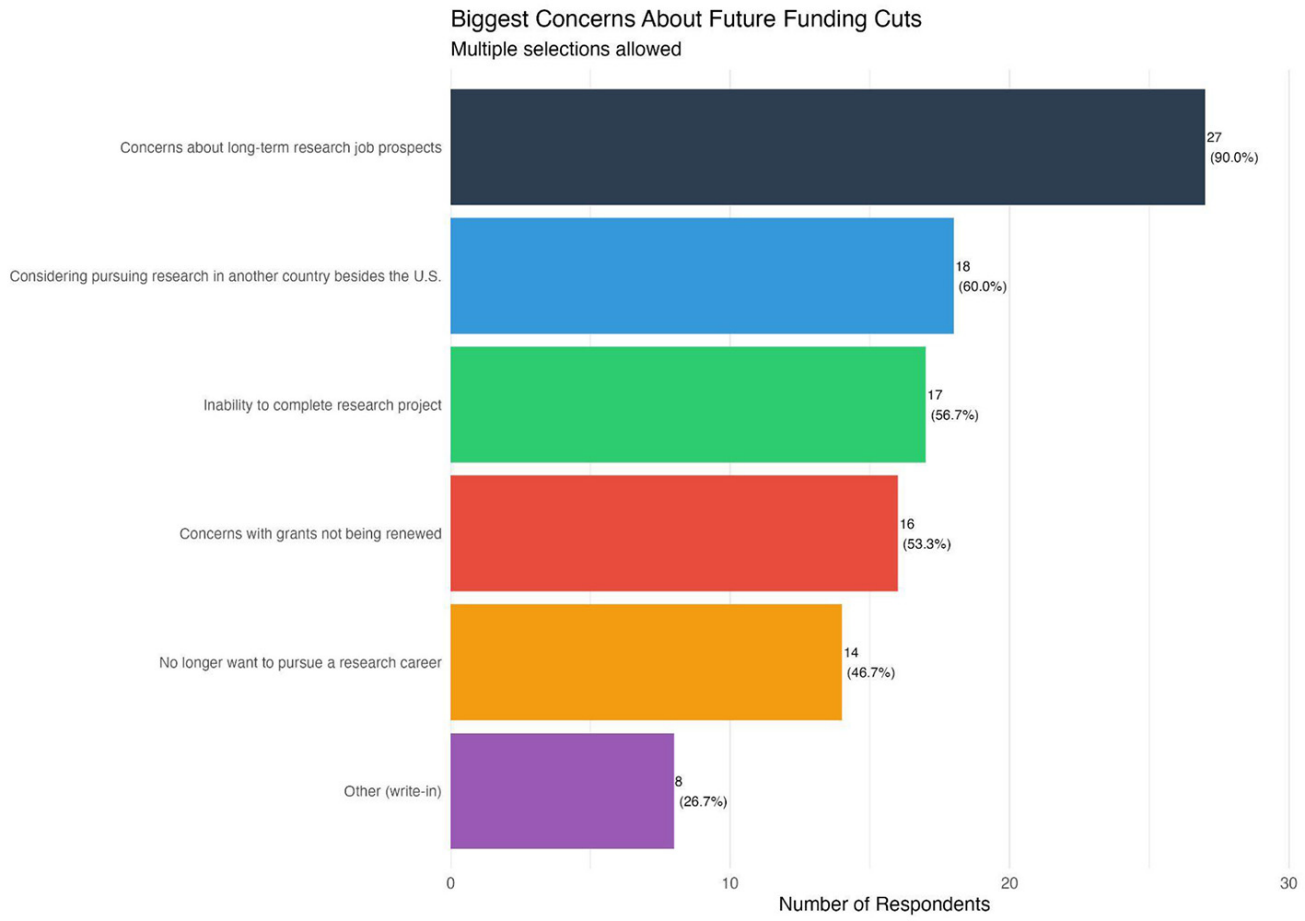
Biggest concerns about future research careers following science funding cuts (Q9; multiple responses permitted). In Survey 2, respondents reported concerns about long-term research job prospects (90.0%), considering pursuing research in another country besides the U.S. (60.0%), inability to complete research project (56.7%), non-renewal of grants (53.3%), no longer wanting to pursue a research career (46.7%), and other (26.7%).

Although the high proportion of “other” responses may indicate important impacts not fully captured by the listed response options, these findings suggest that funding disruptions were associated with consequential professional consequences for respondents. Respondents also identified concerns about the potential future effects of funding cuts on their research and scientific careers (Q9; multiple responses permitted), including long-term research job prospects (90.0%), pursuing research opportunities outside the U.S. (60.0%), inability to complete research projects (56.7%), non-renewal of grants (53.3%), and leaving research careers altogether (46.7%), as well as other concerns (26.7%; Figure 9). These findings raise concern that early career researchers may be discouraged from pursuing long-term scientific careers in the U.S., posing risks to the nation's research enterprise and global competitiveness.

**Figure 10:**
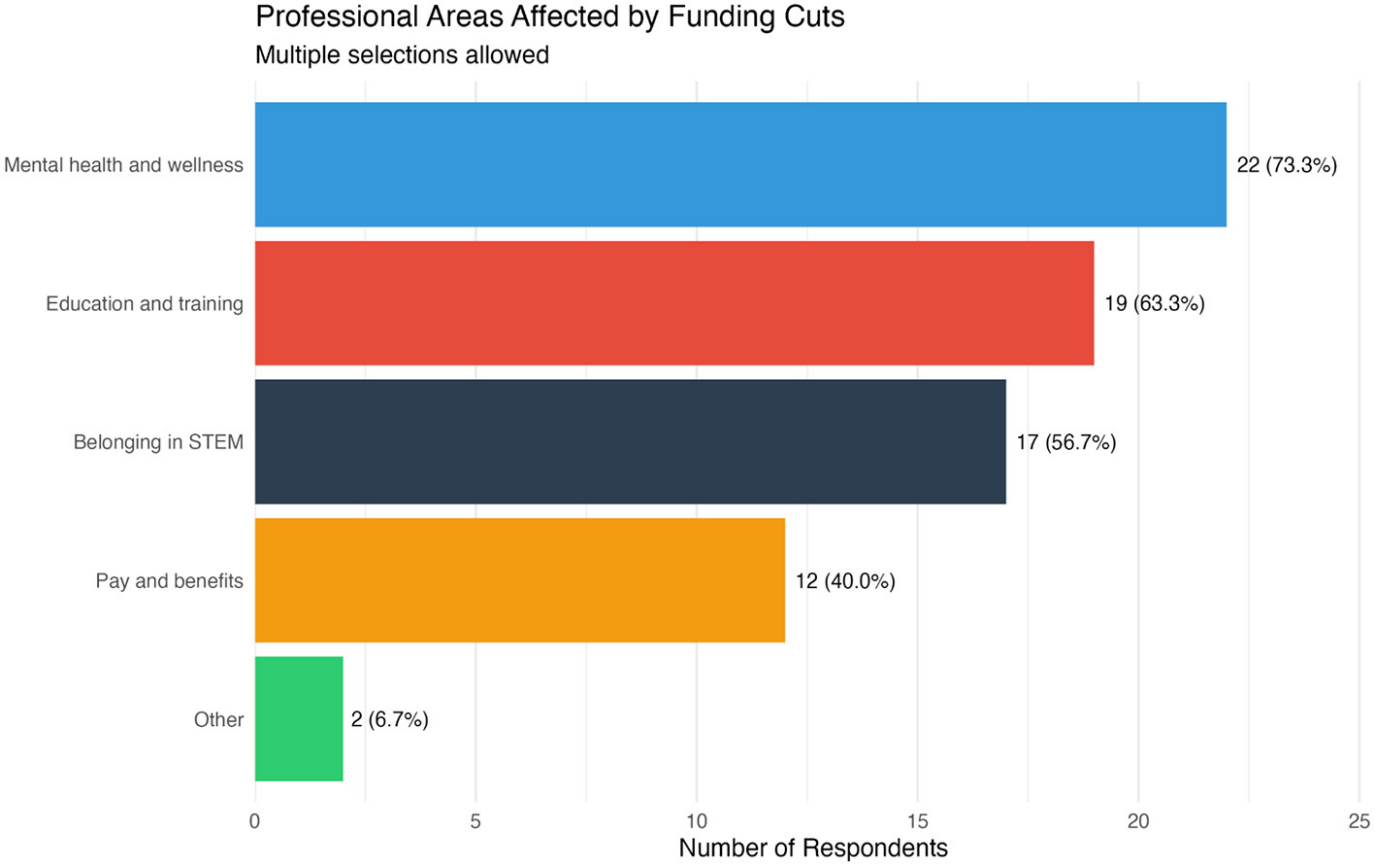
Impacts of federal funding cuts on professional areas of respondents' research and careers (Q10; multiple responses permitted). In Survey 2, respondents indicated systemic issues resulting from federal funding cuts, reporting impacts on their mental health and wellness (73.3%), education and training (63.3%), belonging in STEM (56.7%), pay and benefits (40.0%), and other areas (6.7%).

**Figure 11:**
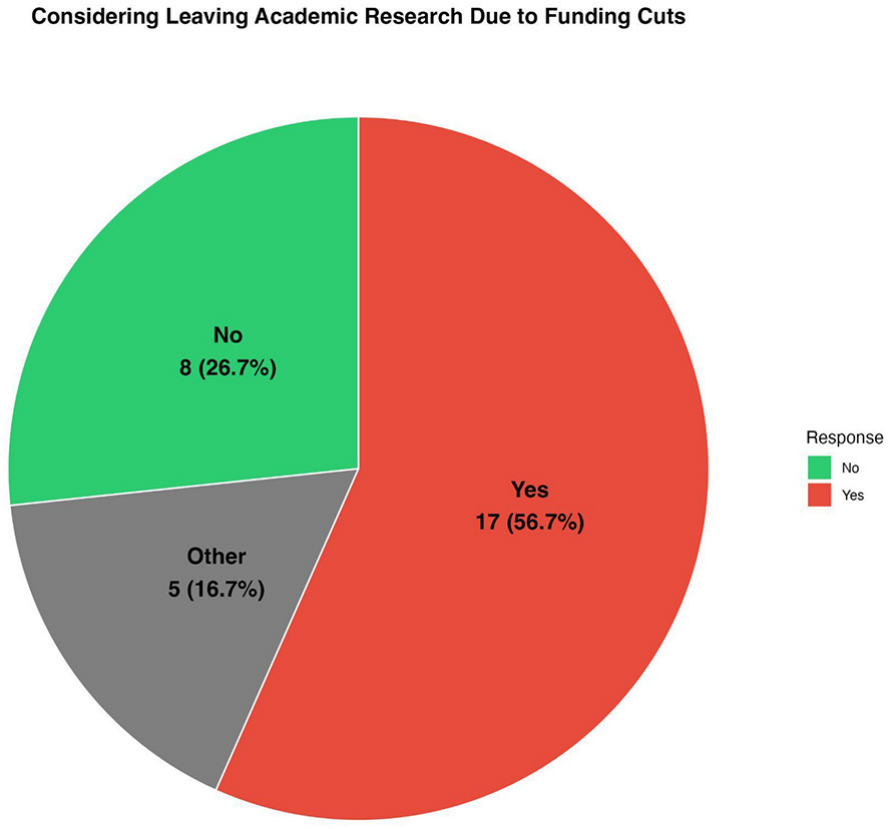
Respondents considering leaving academic research due to science funding cuts (Q11). In Survey 2, the majority of respondents indicated that they were considering leaving academic research (56.7%), while fewer reported that they were not considering leaving (26.7%) or selected “other” (16.7%).

At the systemic level, respondents identified several areas in which federal funding cuts had affected them personally (Q10, multiple responses permitted). The most frequently reported impacts were on mental health and wellness (73.3%), followed by education and training (63.3%), sense of belonging in STEM (56.7%), pay and benefits (40.0%), and other areas (6.7%; Figure 10). When asked whether they were considering leaving academic research (Q11), more than half of respondents answered “yes” (56.7%), fewer answered “no” (26.7%), and some selected “other” (16.7%; Figure 11). These findings suggest that the effects of the Trump administration's actions on federal research funding extended beyond research activities alone, affecting the broader dimensions of professional development and wellbeing that are essential for early career researchers. They also point to systemic pressures that may hinder career progression and contribute to attrition from the U.S. research workforce.

### Perspectives from early career researchers across career stages

In addition to quantitative results, we collected qualitative responses from early career researchers to better represent participant perspectives on how federal policy actions have affected their careers and longer-term futures in science. These responses were drawn from both surveys, with more detailed excerpts included from participants who provided extended reflections.

#### Undergraduate students: lost first research opportunities and barriers to staying in STEM

Although this study primarily aimed to capture the views of graduate students and postdoctoral researchers, undergraduate students also responded, and their perspectives are important to consider because many intend to pursue graduate education and long-term research careers. Reductions in funding for undergraduate research may redirect some students away from academic career pathways in the U.S. or lead them to consider opportunities at universities in other countries (Ruberg, 2025). One notable example of disruption to the research pipeline is the reduction of funding for the NSF Research Experiences for Undergraduates (REU) program, which many students utilize to explore and begin research careers at an early stage (Alonso, 2025a). An undergraduate student from University of Alaska Fairbanks, pursuing a B.S. in wildlife biology and in her 4th year at the time of the survey, stated that her “first undergraduate research project was canceled by [her] university because of federal funding issues despite [her] research being fully funded by the department of veterans.” As a disabled veteran, she also described negative impacts on her mental health, stating that she is physically disabled and calling for the ending of “disability discrimination” in STEM fields within universities. Although this account reflects a single respondent, it illustrates how funding disruptions may intersect with broader concerns about inclusion, accessibility, and persistence in academic research.

#### Graduate students: delayed degrees, canceled grants, and leaving the U.S.

Across disciplines, graduate trainees (National Academies of Sciences, Engineering, and Medicine, 2018) described sudden disruptions to dissertation timelines, job searches, and longer-term career plans following the 2025 federal funding cuts. A neuroscience PhD candidate at Georgetown University reported that “illegal funding cuts at NIH” had delayed his thesis research, and that his research progress in these lost months could not be recovered within a 5-year stipend period. He further explained that the effects were not only logistical, but had also altered his career aspirations. Although he was previously determined to serve his government as a federal scientist, “as an American-born U.S. citizen,” who “once aspired to work for the government as a neuroscientist” he now “desperately want(s) to leave America to pursue science in a different country.” This sentiment was echoed by a graduate student from Morehouse School of Medicine, who completed her PhD in September 2024 and described her prospects as “wide-open” before seeing them disappear “within a few months.” The choice she described—to move into industry or leave the country—exemplifies a broader consequence of the 2025 federal science funding disruptions: an involuntary exodus of newly minted PhDs at the career stage when they might otherwise enter academic pipelines, begin independent research programs, and create opportunities to mentor the next generation of scientists in the U.S. (Barbosu, 2025). When asked about the future of science, she said “it is very disheartening” that she “worked so hard for so long for the chance to make a difference in science, and a political agenda is decidedly in [her] way when just trying to help people is NOT a political issue.”

Separately, a PhD student from University of Virginia described being selected for a position at Oak Ridge National Laboratory, which was affected by “required freeze hiring due to budget cuts and resulting shortfalls.” He also cited canceled funds that would have supported his work, including an Environmental Protection Agency (EPA) grant that would have allowed him to “use one semester's worth of funding to take the time needed to wrap up [his] research and/or give [him] some buffer to figure out next steps.” These accounts suggest that federal funding disruption has affected graduate trainees across universities and national laboratories, limiting access to training opportunities and disrupting progression along research career pathways. Consistent with our quantitative survey results, these responses further suggest that sustained federal funding uncertainty may contribute to early career researchers seeking research and other professional opportunities outside the U.S., with negative consequences for the U.S. research enterprise.

#### Postdoctoral fellows: grant terminations and unstable paths to independence

If graduate students experienced the derailing of their careers, postdoctoral fellows described the bridge to independence and their future in science as increasingly unstable. Postdoctoral researchers (National Academies of Sciences, Engineering, and Medicine, 2014) occupy a narrow and often precarious position between trainee and independent investigator. As a result, research funding disruption can affect both their livelihoods and pathways to faculty positions. In response to our survey, a postdoctoral researcher at Emory University stated that “these drastic and arbitrary cuts to science funding hurt early career folks the worst.” She described how her NIH-funded IRACDA fellowship was terminated because its DEI mandate “no longer align(ed) with NIH priorities.” The decision to cut IRACDA program funding not only revoked her salary, but also more broadly discourages diverse faculty from remaining in the pipeline (Rybarczyk et al., 2016). By eliminating teaching opportunities at Minority Serving Institutions (MSIs) such as Spelman College due to these funding cuts, we are hindering our nation's ability to continue broadening participation in STEM disciplines. When asked about her views on the broader future of science, the postdoctoral researcher stated that “the future of science looks bleak,” but that she is “trying to be hopeful that sustained advocacy and activism will be successful and improve the outlook.” Similarly, at Arizona State University, a postdoctoral researcher recounted the abrupt cancellation of her NSF grant in the middle of a very fulfilling role, a project “terminated early, not because it lacked value or impact, but because it no longer aligned with shifting federal priorities around equity and public-interest tech.” As to how this cancellation made her feel, the postdoctoral researcher shared that “it was crushing—not just because I lost my role, but because the work mattered.” Overall, the loss of such program funding threatens to turn away valuable talent from our research enterprise.

### Response strategies among early career researchers and their institutions

We collected quotes and narratives from early career researchers, including advocacy actions taken in response to the Trump administration's actions on federal science funding (Survey 1). Reported actions included sharing experiences of federal research funding cuts with peers, mentors, and professional networks; learning how to advocate for scientific research; contacting elected representatives; educating the public; and attending or organizing protests and related events. We then grouped these advocacy actions into broader categories to facilitate review of the responses (Table 2).

**Table 2:** Response strategies reported by survey 1 respondents.

Response strategies quotes	Category
“After the termination of my NSF funded project and job, I have been sharing my experience with peers, mentors, and professional networks to raise awareness about how these funding cuts impact (early career) researchers and disrupt ethically grounded, community-engaged work.”	Communication
“While I haven't engaged in formal policy advocacy yet, I believe in speaking openly about these impacts and continue to look for opportunities to make our collective voices heard.”	Communication
“My advocacy actions have been learning about the scope of the issue from other scientists, thinking of how to explain it to the general public, and using my voice to educate the people around me.”	Learning to advocate, communication
“I have attended multiple protests. I am also hoping to both conduct and help organize events through Stand Up For Science.”	Protests, events
“I have called and written [to] my representatives and urged friends and family to do the same.”	Contacting representatives

Respondents in Survey 1 self-reported advocacy actions they had taken in response to the Trump administration's actions on federal science funding. These responses were then grouped into broader thematic categories for analysis.

**Figure 12:**
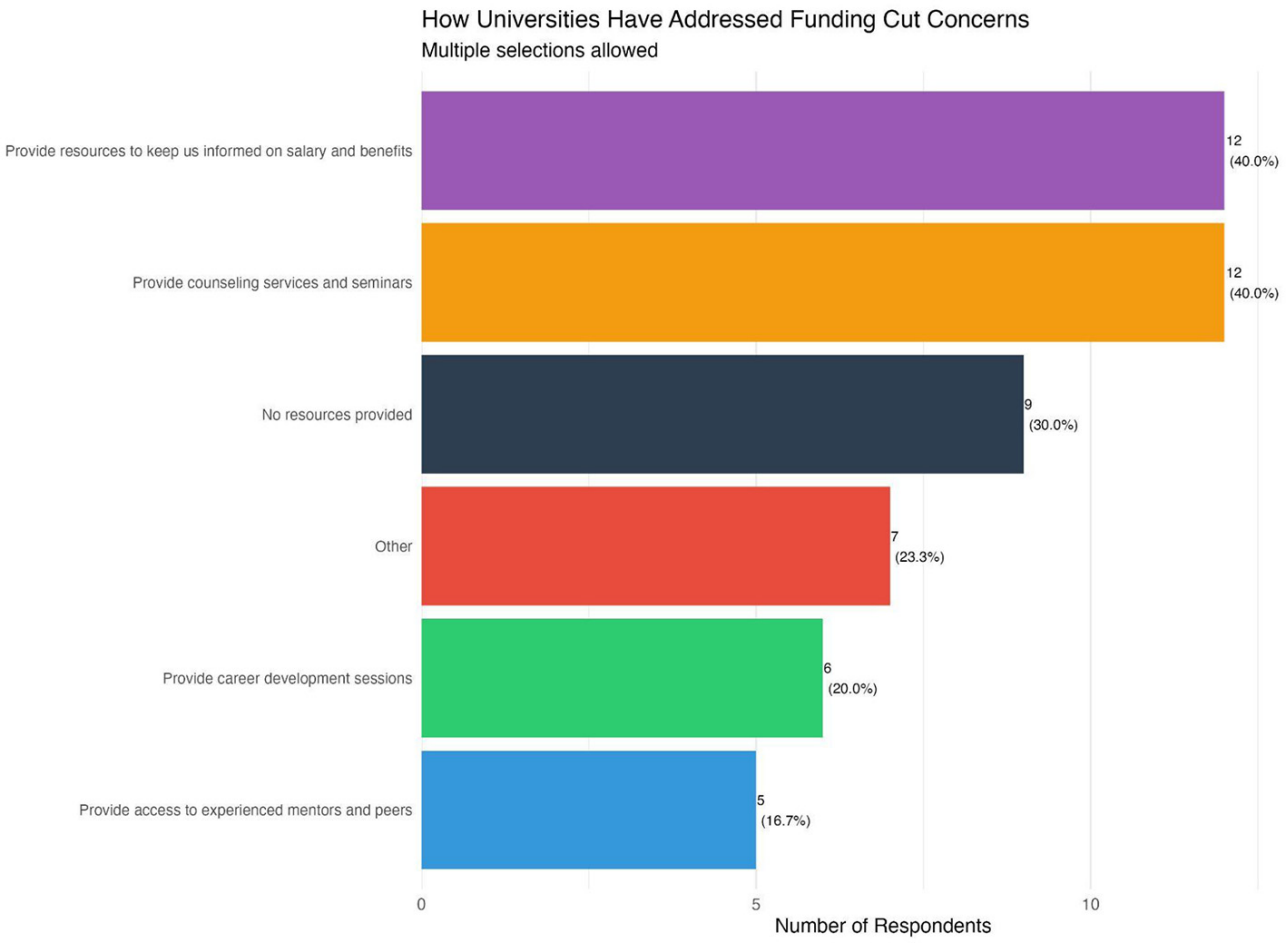
University responses to professional concerns related to federal research funding cuts (Q12; multiple responses permitted). In Survey 2, respondents most frequently reported that their university provided resources to keep them informed about salary and benefits (40.0%), and counseling services and seminars (40.0%), followed by no resources (30.0%), other forms of support (23.3%), career development sessions (20.0%), and access to experienced mentors and peers (16.7%).

When asked how their institution had constructively addressed professional concerns related to federal funding cuts affecting their research (Q12; multiple responses permitted), respondents most frequently reported resources to keep them informed about salary and benefits (40.0%), and counseling services or seminars (40.0%), followed by no resources (30.0%), “other” forms of support (23.3%), career development sessions (20.0%), and access to experienced mentors and peers (16.7%; Figure 12). These findings highlight the range of institutional responses to funding-related concerns, and underscore the importance of support mechanisms for sustaining research activity and promoting positive working environments for early career researchers during periods of disruption such as the second Trump administration.

Conversely, when asked in an open-ended question what Congress could do to reduce the fallout from funding cuts (Q13), respondents emphasized the need for sustained federal funding, particularly for NIH and NSF. Their responses highlighted the importance of protecting and stabilizing this funding, communicating its broader value, supporting alternative funding models and initiatives, and ensuring adequate staffing of the federal workforce responsible for administering these grants (Table 3). These quotes suggest that respondents view the legislative branch as a critical actor in addressing the challenges faced by early career researchers during a period of reduced funding for the federal science agencies under the Trump administration.

**Table 3:** Respondent recommendations for congressional action to support early career researchers (Q13).

Congressional action quote	Category
“Do not cut the NIH or NSF budgets. Do not mess with grants that are already there.”	Funding protection
“Protect funding for science and prevent delays in distributing funds.”	Funding protection
“Ensure the stability of federal funding for the sciences.”	Funding stability
“Reverse cuts to indirect rates [at NIH].”	Funding stability
“Create dual track of funding mechanisms: (1) Fund basic research that fits into societal need; (2) Fund research that has the potential on commercialization.”	Funding models (dual track)
“Congress can commit to funding student fellowships and fund NIH to examine social determinants of health.”	Funding models (fellowships)
“Fund diversity initiatives, support underrepresented scholars, support climate, health, environmental justice, sex differences, trans health.”	Funding initiatives (including DEI)
“We need Congress to fund national science agencies at their full budgets and not allow DOGE or science agencies to cut funds capriciously.”	Funding restoration
“Listen to scientists, give the scientific community our funding back!”	Funding restoration, communication
“Platform the voices of scientists, patients, and community advocates directly impacted by these cuts, and act as advocates for them in appropriations settings, etc.—speak loud and publicly about these issues.”	Communication
“Provide retroactive support for staff that were illegally fired [from NIH].”	Staffing federal workforce

Representative quotes from respondents to an open-ended question in Survey 2 were then grouped into thematic categories based on recurring recommendations (e.g., funding protection, funding stability, and communication). Many responses emphasized the need for sustained federal support for NIH and NSF.

## Study limitations

This study should be interpreted in light of several limitations. First, the small sample size limits generalizability. The survey was only open for a brief period of time, making this a rapid-response pilot study designed to capture participant perspectives while the Trump administration's actions on federal science funding were ongoing and their effects on early career researchers were especially acute. The goal of this work was to document how early career researchers who chose to respond were affected in terms of their research projects and career trajectories, and how they were navigating these changes in real-time during that timeframe. Accordingly, while the findings should be interpreted as illustrative rather than nationally representative, they provide timely insight into the lived experiences of early career researchers during a period of substantial national policy change.

Second, while we did not select for particular groups in terms of demographics and disciplines, the respondents to our surveys were predominantly White PhD students from R1 and public institutions in the life sciences who reported being substantially affected by the Trump administration's actions on federal science funding. This distribution may reflect several factors, including the professional and organizational networks through which the survey circulated, differential participation across fields and institution types, and the possibility that individuals who were more acutely impacted were more likely to respond. As a result, the findings should not be interpreted as representative of all early career researchers across disciplines, institutional settings, or demographic groups. More specifically, the predominance of PhD students and respondents in the life sciences may reflect both the structure of the early career STEM workforce and the particular networks through which the survey was disseminated. This interpretation is consistent with national patterns in U.S. academic research, where biological and biomedical sciences have remained the largest doctoral field as of 2022 (Smith et al., 2024), biological and biomedical sciences plus clinical medicine accounted for 54.5% of postdoctoral employment as of 2023 [National Center for Science and Engineering Statistics (NCSES), 2025] and the life sciences accounted for 57% of higher-education R&D spending as of 2023 (National Science Board, National Science Foundation, 2025). It is also consistent with the concentration of doctoral training and postdoctoral employment at research-intensive institutions: in FY 2021, institutions with very high research activity accounted for nearly 75% of U.S. academic R&D spending, enrolled about 80% of science and engineering doctoral students, and employed over 80% of science and engineering postdocs. Public universities accounted for 65% of academic R&D spending and just over half of postdocs were located at these institutions (National Science Board, National Science Foundation, 2023).

Relatedly, although some groups were underrepresented in the sample, the present study does not allow us to determine why this was the case. It is possible that some early career researchers, including those from minoritized groups or from more vulnerable institutional settings, may have been less likely to participate during a period of heightened political uncertainty. However, this remains speculative, and we cannot draw firm conclusions about nonresponse patterns from the available data. For this reason, caution is warranted in interpreting the demographic composition of the sample beyond describing it. Finally, this study was intentionally focused on the sciences, including the social sciences, and was not designed to assess the effects of federal funding disruptions across the full higher education landscape. Humanities fields may be affected through different funding structures, institutional arrangements, and advocacy channels, and are therefore outside the scope of the present analysis. This narrower focus does not diminish the importance of those fields, but it does define the boundaries of what this study can claim. Future work would benefit from larger and more diverse samples, longer data collection windows, and broader disciplinary coverage in order to better assess how funding disruptions are being experienced across the wider research and higher education enterprise.

## Discussion

### Federal science funding cuts are disrupting early career pathways

Our survey results clearly showed the detrimental impacts of the Trump administration's actions on early career researchers at various career stages, both in terms of data collection and personal narratives elaborated upon by the answers of respondents. In this section, we discuss the survey responses in a broader context and suggest potential interpretations of the results as it relates to the current and future state of the research enterprise and workforce. The vast majority of basic scientific research occurs at academic institutions, and the lion's share of research funding has historically come from the U.S. federal government (Borges, 2025). Our analysis captured an important segment of the STEM workforce in the early career population that was grappling in real-time with drastic cuts to federal research funding and the impacts of these cuts on their research and careers trajectories, and beyond. Examples of these disruptive actions on early career scientists in particular, include removing the ability of second year graduate students to apply to the NSF GFRP (Langin, 2025) and the fact that funding for TRIO programs was set to be completely eliminated in President Trump's FY 2026 budget proposal (Alonso, 2025b), as a few examples. These measures have discouraged talented trainees from contributing to the scientific enterprise and our global competitiveness.

### Structural vulnerability shapes exposure to funding cuts

These actions have resulted in various impacts on universities across the nation, including likely those from our respondents working at different types of institutions which we noted after the fact, although did not explore this question in depth. One could speculate that impacts of the funding cuts may be different depending on the type of institution where the respondents are currently studying and working, although that would constitute a broad generalization. However, the higher education community is well-aware of these varied impacts based on how research is funded at private vs. public institutions, as well as R1s vs. R2s vs. RCUs, so in general future analyses of this issue may be warranted in order to further address impacts of the Trump administration's actions on the research enterprise and STEM pipeline, and the large pool of talent driving scientific progress forward. However, given the targeting of minoritized groups by the Trump administration (Boyd, 2025) causing scientists to have to remove DEI-related words from grants (Randazzo et al., 2025), we can speculate that, although early career researchers from certain types of institutions (e.g., non-R1 universities) or those belonging to various minoritized groups in the sciences may have felt apprehensive to respond to the surveys, many may have wanted to either express their views openly in an unbiased manner and did not do so for various reasons including potential retaliation. Nevertheless, our analysis captured an important segment of early career respondents in life sciences, a large fragment of the STEM research workforce in addition to other disciplines (National Science Board, National Science Foundation, 2021), during a time when science was severely under attack.

Across predoctoral and postdoctoral stages, these accounts from early career survey respondents revealed recurring patterns. Rather than describing only a temporary budget constraint, respondents expressed broader concerns about whether the U.S. still offers a viable, stable, and values-aligned environment in which to build long-term scientific careers. Without targeted interventions like bridge-funding and visa-retention incentives, the U.S. risks hollowing out the very STEM workforce on which its future discovery and global competitiveness depends, particularly at a time of intensifying competition from China, which now surpasses the U.S. in total peer-reviewed research output, has rapidly narrowed the gap in overall research and development investment, and has been the world's largest producer of STEM doctorates since 2007 (Hourihan, 2026; Langston, 2025; Long, 2025).

Postdoctoral researchers who discussed their experiences with program funding cuts acknowledged that better support for early career researchers and stable investment in science is needed, in order to prevent the narrowing of talent in the biomedical research workforce across institutions. This is especially important for institutions whose primary goal is to broaden participation. In that sense, there are two systemic vulnerabilities affecting postdoctoral fellows. First, postdoctoral researchers often have few safety nets, and when a single award disappears, entire research trajectories—and the possibility of a scientific career—can disappear with it. This can be especially true at under-resourced institutions such as Historically Black Colleges and Universities (HBCUs), Minority Serving Institutions (MSIs), and Tribal Colleges and Universities (TCUs), which often rely more heavily on federal funding and have fewer alternative funding streams than wealthier research-intensive institutions. In such cases, federal research funding cuts can threaten not only individual careers but also the institution's financial stability, operational capacity, and long-term viability (Williams and Davis, 2019; Colorado, 2024; Nelson and Frye, 2016). Second, some of the projects most exposed to political shifts were equity focused, public-interest, or diversity-related projects, including work serving marginalized populations (Cooper, 2025). The disruption of these lines of inquiry may have broader consequences for the U.S. research enterprise, particularly given evidence that diversity in scientific collaboration is associated with greater scientific impact and that underrepresented scientists often generate especially novel contributions (Palmer, 2025b). These opportunities for research innovation are the ones the U.S. can least afford to lose.

Indeed, we found that many early career respondents noted significant impacts of the funding science cuts on their experiences within the institution, and broader professional trajectories, suggesting the need to address their inability to undertake and complete research projects due to the uncertainty in federal grant funding. As a result, respondents would desire to abandon the field of scientific research altogether. Additionally, the Trump administration's impacts would not only derail scientific careers for talented researchers, but these funding cuts send a detrimental cascading effect throughout academia including for a number of systemic issues. In this sense, our results show that the lack of support (both financial and otherwise) for early career scientists from both institutions and Congress, can significantly hinder their professional trajectories and desire to remain in the U.S. for research studies and work (Aksenfeld and Ahart, 2025).

When collecting demographic information from respondents, this was an optional question that all respondents chose to address, perhaps signifying a desire to highlight their views in response to the Trump administration's actions given the targeting of certain populations in the sciences. Responses showed a large percentage of White individuals which is notable, as academics from historically marginalized groups may be apprehensive from responding to such questions (Kassam, 2025), potentially due to fear of the Trump administration's actions on their professional future, whether or not this impacts them and/or their institution. Alternatively, many early career researchers who responded to the survey shared quotes, stories and advocacy actions they have taken, and these surveys could likely provide an additional outlet for expression for those that may have wanted to speak out but may not have had other avenues to do so at that time outside of what we provided for them to respond, and this could have been presumably appreciated given that SRC is not affiliated with any given institution eliminating some of the barriers.

Beyond the individual responses, universities themselves may have also been impacted as a whole, which we did not address in depth. On the flip side, early career researchers likely looked to their institutions to help address some of the issues they were facing based on the Trump administration's actions on the research enterprise. Based on answers reported by survey respondents, their universities have taken measures to address these issues, and perhaps the responses obtained could be useful as the ecosystem seeks to rebuild from this chaos by providing additional support and/or different types of resources for early career scientists. In addition to the university angle, Congress plays an important role in supporting the next generation of researchers, and opinions expressed by our respondents suggest potential actions that could be taken in the legislative branch to support the future of science. These could include ways to address broad systemic issues in academic research and grant funding to help early career scientists feel more supported in the current environment and moving forward, and researchers should communicate why this funding is critical to the enterprise with their Members of Congress.

### Science funding cuts are catalyzing advocacy and collective mobilization

In response to the Trump administration's detrimental actions on the research enterprise, scientists have been fighting back through various mechanisms (Garisto et al., 2025), and this survey is part of this phenomenon. While many players are involved, a number of organizations have stepped up significantly to provide assistance to early career researchers including through establishing funding mechanisms to bridge the gap (American Chemical Society, 2025; O'Brien, 2025). Additionally, it is also important to note that all over the nation, early career researchers came together across disciplines, institutions, and from multiple locations and career stages to fight back and reimagine their future in science. Some examples of activities undertaken, which our survey alluded to when asking about advocacy actions taken by early career scientists, have included campus walkouts and rallies across the country documented in various outlets (Berntsen et al., 2025), and these have been loud and clear public declarations that participating in science is not only a national asset but a democratic right. These efforts led to important initiatives such as the launch of early career advocacy networks and coalitions that have emerged on major campuses and have been profiled on the national stage including at conferences and elsewhere (Scientist Network for Advancing Policy, 2025). They have also collected local newspaper op-eds and personal testimonies from scientists across disciplines, in order to put faces to the futures of those affected by science funding cuts. Together, these efforts throughout the nation, which our survey also addressed, helped transform early career scientists from isolated trainees into a coordinated advocacy force.

### Supporting early career researchers is essential to the future of the U.S. research enterprise

Support in the form of grant funding for early career researchers and their careers in science is imperative to continue in a manner that is robust and sustained for the long-term, beginning with the current environment and into the future. This funding can ultimately help the U.S. develop a strong STEM workforce and elevate our global competitiveness. While a number of players have a responsibility to act on achieving this goal, perhaps in unison, trainees themselves have taken actions for their own future in science as described briefly in our survey. We emphasize that early career scientists—who have the most to lose and the least institutional power—remain at the forefront of both the fallout and the resistance, and their involvement is critical for the future of STEM. This includes researchers from different backgrounds, who contribute to our scientific innovation and economy and are often most vulnerable in academia, and especially following the Trump administration's actions to dismantle the entire enterprise. Yet if this moment revealed anything, it's that scientists are not only researchers—they are organizers, storytellers, coalition-builders, and defenders of democracy, and that early career scientists have a voice that matters in the process that should be heard loud and clear by our federal decision-makers. Thus, what began as a reactive moment has now become a generative one by and for early career scientists and those who support them: a movement for a future where science is inclusive, accountable, and deeply rooted in the public good, and one that we can and should all participate in for our nation's prosperity and leadership.

## Data Availability

The raw data supporting the conclusions of this article will be made available by the authors, without undue reservation.
