# Supplementary material for: Early career researcher responses to 2025 United States federal science funding disruptions: a rapid-response pilot survey
Source: Front Sociol. 2026 Jul 20;11:1790840. doi: 10.3389/fsoc.2026.1790840 (PMC13450147; doi:10.3389/fsoc.2026.1790840)
Supplement: Supplementary file 1 [file Supplementary_file_1.docx]

**Supplementary Appendix.** Survey questions asked during online surveys using Google Forms between May 23 and June 6, 2025.

**Survey 1: Stories survey: Collecting early-career researcher quotes and stories on impacts resulting from federal science funding cuts**

Email:

First and last name:

Academic institution:

Quote or story (250 words):

Advocacy action taken (250 words):

Other comments you would like to share (optional):

**Survey 2: Impacts of federal science funding cuts on early-career researchers**

Q1. What is your career stage?

- Undergraduate student
- PhD student
- Master’s student
- Postdoctoral researcher
- Other

Q2. How long have you been in your current position?

- Less than 1 year
- 1-2 years
- 2-3 years
- 3-4 years
- 5+ years
- Other

Q3. Which academic institution are you currently working in? (write-in)

Q4. What is your field of study?

- Life sciences
- Environmental sciences
- Engineering
- Social sciences
- Other

Q5. Which of the following best describes your racial identity? (optional)

- American Indian or Alaska Native
- Asian
- Black or African American
- Native Hawaiian or Other Pacific Islander
- White
- Bi or Multi-racial
- Other

Q6. Which federal agency is currently funding your research? (multiple options can be checked)

- NIH
- NSF
- DOE
- NASA
- USDA
- NIST
- DOD
- Other

Q7. On a scale of 1 - 10, how much have the recent funding science cuts affected you professionally? 1= No effect, 5= Moderately affected, 10= Severely affected

Q8. Which of the following impacts have you experienced?

- University froze hiring and/or rescinded the offer
- My stipend and benefits were cut or delayed
- Nothing has changed for me
- Other response

Q9. What is your biggest concern on potential future funding cuts impacting your research and career in science? (multiple options can be checked)

- Inability to complete research project
- Concerns with grants not being renewed
- No longer want to pursue a research career
- Concerns about long-term research job prospects
- Considering pursuing research in another country besides the U.S.
- Other

Q10. In what way have federal funding cuts affected you professionally in terms of opportunities in the following areas? (multiple options can be checked)

- Education and training
- Belonging in STEM
- Mental health and wellness
- Pay and benefits
- Other

Q11. Are you considering leaving academic research as a whole due to the funding cuts?

- Yes
- No
- Other

Q12. Has your university addressed your professional concerns as related to the funding cuts? If yes, please select your options below.

- Provide career development sessions
- Provide access to experienced mentors and peers
- Provide counseling services and seminars
- Provide resources to keep us informed on salary and benefits
- Other
- No resources provided

Q13. What can Congress do in order to support your future in research? (write-in)
