# Supplementary material for: Early career researcher responses to 2025 United States federal science funding disruptions: a rapid-response pilot survey
Source: Front Sociol. 2026 Jul 20;11:1790840. doi: 10.3389/fsoc.2026.1790840 (PMC13450147; doi:10.3389/fsoc.2026.1790840)
Supplement: Supplementary file 2 [file Table_1.docx]

**Supplementary Table 1. Representative Organizations for Survey Outreach.** Outreach was broadly conducted through our networks to a broad range of stakeholders including universities, non-profits and other organizations. Within these organizations, we targeted specific categories of expertise, including career development, research,

policy, and STEM pipeline diversification. For some of these entities, publication authors had existing connections. The table includes a representative sampling of the organizations with high levels of early career membership,

and/or those that have strongly supported early career scientists both in the Trump administration and more generally, allowing us to reach the target populations while keeping this outreach broad.

| **Type of Organization** | **Category** | **Representative Organizations** |
| --- | --- | --- |
| Non-profit | Career development | National Postdoctoral Association (NPA), Graduate Career Consortium (GCC) |
| Non-profit | Career development | Society for Advancement of Chicanos/Hispanics & Native Americans in Science (SACNAS) |
| Non-profit | Policy | National Science Policy Network (NSPN), Engineers and Scientist Acting Locally (ESAL) |
| University | Research | University of California Campuses, Howard University, George Washington University, Yale University, Massachusetts Institute of Technology |
| University program | Diversity | Louis Stokes Alliance for Minority Participation Program, Meyerhoff Graduate Fellows Program |
| Association program | Policy | American Association for the Advancement of Science (AAAS) CASE, State fellowship programs |
| Association | Higher education | Association of American Universities (AAU), Association of Public and Land-Grant Universities (APLU) |
| Scientific society | Research | American Chemical Society (ACS), Genetics Society of America (GSA), Sigma Xi |
